# Comparative Proteomics of Rubber Latex Revealed Multiple Protein Species of REF/SRPP Family Respond Diversely to Ethylene Stimulation among Different Rubber Tree Clones

**DOI:** 10.3390/ijms18050958

**Published:** 2017-05-02

**Authors:** Zheng Tong, Dan Wang, Yong Sun, Qian Yang, Xueru Meng, Limin Wang, Weiqiang Feng, Ling Li, Eve Syrkin Wurtele, Xuchu Wang

**Affiliations:** 1 Institute of Tropical Biosciences and Biotechnology, Chinese Academy of Tropical Agricultural Sciences, Haikou 571101, China; tongzheng@itbb.org.cn (Z.T.); wangdanqz2009@126.com (D.W.); sunyong_03119308@126.com (Y.S.); yangqianhaikou@163.com (Q.Y.); xuerumeng1203@126.com (X.M.); wanglimin9696@163.com (L.W.); 2 College of Agriculture, Hainan University, Haikou 570228, China; fengweiqiang678@163.com; 3 Department of Genetics, Development and Cell Biology, Iowa State University, Ames, IA 50011, USA; liling@iastate.edu (L.L.); evewurtele@gmail.com (E.S.W.); 4 Center for Metabolic Biology, Iowa State University, Ames, IA 50011, USA; 5 Department of Biological Sciences, Mississippi State University, Starkville, MS 39762, USA

**Keywords:** comparative proteomics, *Hevea brasiliensis*, ethylene stimulation, latex productivity, rubber elongation factor, small rubber particle protein

## Abstract

Rubber elongation factor (REF) and small rubber particle protein (SRPP) are two key factors for natural rubber biosynthesis. To further understand the roles of these proteins in rubber formation, six different genes for latex abundant REF or SRPP proteins, including REF_138,175,258_ and SRPP_117,204,243_, were characterized from *Hevea brasiliensis* Reyan (RY) 7-33-97. Sequence analysis showed that REFs have a variable and long N-terminal, whereas SRPPs have a variable and long C-terminal beyond the REF domain, and REF_258_ has a β subunit of ATPase in its N-terminal. Through two-dimensional electrophoresis (2-DE), each REF/SRPP protein was separated into multiple protein spots on 2-DE gels, indicating they have multiple protein species. The abundance of REF/SRPP proteins was compared between ethylene and control treatments or among rubber tree clones with different levels of latex productivity by analyzing 2-DE gels. The total abundance of each REF/SRPP protein decreased or changed a little upon ethylene stimulation, whereas the abundance of multiple protein species of the same REF/SRPP changed diversely. Among the three rubber tree clones, the abundance of the protein species also differed significantly. Especially, two protein species of REF_175_ or REF_258_ were ethylene-responsive only in the high latex productivity clone RY 8-79 instead of in RY 7-33-97 and PR 107. Some individual protein species were positively related to ethylene stimulation and latex productivity. These results suggested that the specific protein species could be more important than others for rubber production and post-translational modifications might play important roles in rubber biosynthesis.

## 1. Introduction

Approximately 2500 plants generate natural rubber latex, but *Hevea brasiliensis* is the only widely used rubber tree for natural latex production [1]. Natural rubber biosynthesis is synthesized in the specialized cells which have a cytoplasm called latex [2]. In *H. brasiliensis*, these specialized cells are articulated laticifers, and are located within a thin layer under the tree bark. Natural latex contains ~35% rubber particles, ~60% water, and a small amount of lipids, carbohydrates, and lots of proteins [3,4].

Natural rubber polymer is found in rubber particles enclosed by a contiguous, monolayer biomembrane in the laticifer cells [5,6]. Rubber biosynthesis includes three major processes: the synthesis of the rubber monomer isopentenyl pyrophosphate (IPP); the synthesis of initiator molecules such as geranyl pyrophosphate, farnesyl pyrophosphate, and geranylgeranyl pyrophosphate (APPs); and the elongation of isoprene polymers. The synthesis of IPP and APPs is common to all plants [7]. However, the elongation of these subunits into rubber in latex-producing plants occurs at the surface of the rubber particles and is catalyzed by many protein complexes [8,9]. An experiment incubating the extracted rubber particles with [1-^14^C]-IPP confirmed the production of rubber polymer from IPP by the particles [5,9,10].

Although several candidates for rubber biosynthetic proteins have been identified through their association with rubber particles and in vitro biosynthesis rubber assay, the process of rubber biosynthesis is far from being understood. Two related classes of proteins have been shown to play a role in natural rubber biosynthesis: one is rubber elongation factor (REF) [11], the other is small rubber particle protein (SRPP) [12]. *Hevea* laticifers are layers of contiguous cells that are formed parallel to the vascular cambium [13]. Immunogold staining showed that REF is localized in both large and small rubber particles and in all laticifer layers, but SRPP is predominantly localized in small rubber particles and in laticifer layers in conducting phloem [14]. Computational study predicted that REF protein had organized aggregates of β-sheet, whereas SRPP protein formed helical fold structures [15]. Both REF and SRPP are highly hydrophobic proteins, however, they exhibit different affinities for the monolayer of rubber particle, and ellipsometry experiments showed that REF seems to penetrate into the rubber particle membrane while SRPP binds on the membrane surface [15,16].

REF and SRPP share a common REF domain. Studies on REFs and SRPPs in *H. brasiliensis* have been focused on a 14.6 kDa REF (gi|132270) and a 24 kDa SRPP (gi|14423933); their precise roles in natural rubber biosynthesis are still unknown. To better understand the roles of REF and SRPP in rubber biosynthesis, in this research, we characterized the six genes coding for the most abundant REF/SRPP proteins in latex from *H. brasiliensis* RY 7-33-97, compared their protein sequence structures and cellular locations, then investigated their expression pattern in response to ethylene at both the mRNA and the proteomics levels, and finally compared their abundance among three rubber tree clones with different levels of latex productivity. The results revealed that all the REF/SRPP proteins all have multiple protein species, but only a few protein species responded positively to ethylene stimulation and related to the latex productivity of rubber tree clones.

## 2. Results

### 2.1. REF and SRPP Subfamily Members in H. brasiliensis

Rubber elongation factor (REF) and small rubber particle protein (SRPP) are two key factors for natural rubber biosynthesis. A total of 18 REF/SRPP like sequences were predicted from the genome of *H. brasiliensis* after genomic sequencing [17]. In addition, in our previous work, we found the 13 of the 18 sequences coding protein can be found in rubber latex by shotgun analysis [18], and only 6 of them were abundant. Based on this information, the sequences of the six REF/SRPP genes were confirmed in *H. brasiliensis* by cDNA clone, and named as *REF_138_*, *REF_175_*, *REF_258_*, and *SRPP_117_*, *SRPP_204_*, *SRPP_243_* based on their protein length. *REF_138_*, *REF_175_*, *SRPP_117_*, *SRPP_204_* and *SRPP_243_* are identical to the predicted sequences by genome, but *REF_258_* has a much longer N-terminal than the predicted one.

**Figure 1:**
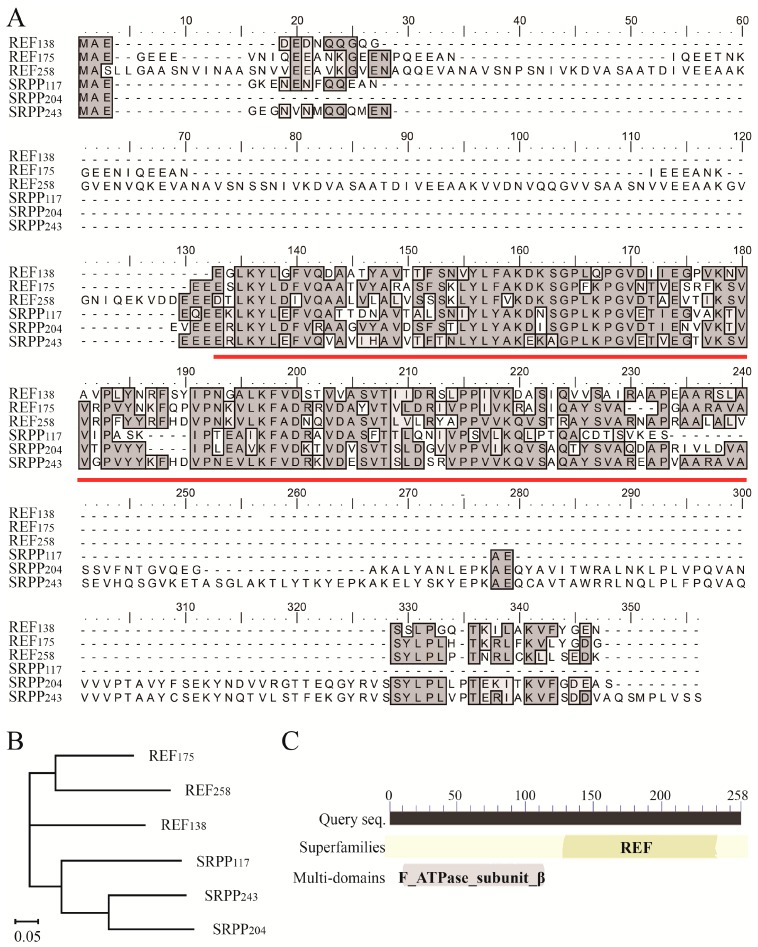
Multiple protein sequence alignment and phylogeny analysis of different REF/SRPP family members in the rubber tree. (**A**) Sequence alignment of REF and SRPP. Threshold into gray frames is 50% and the approximate location of the REF domain is underlined. Identical and similar amino acids are colored with dark and light gray frames, respectively; (**B**) Phylogeny relationships of REF and SRPP by complete protein sequence. Branch lengths are proportional to accumulated amino acid substitutions. Accession numbers in GeneBank of the six genes are listed in the Materials and Methods section; (**C**) The multiple domains of REF_258_.

Sequence alignment of the six REF and SRPP proteins from *H. brasiliensis* RY 7-33-97 showed the relatively conserved REF domain [19]. The conserved REF domain is ~110 amino acid (aa) in all proteins except for SRPP_117_, which has a small deletion (Figure 1A). Other than the REF domain, the N- and C-terminal of the six REF and SRPP members have clear differences in length and compositions of amino acids (Figure 1A). REF subfamily members have a short C-terminal of constant length but an N-terminal of variable length, while SRPP subfamily members have a short N-terminal but a C-terminal of variable length. REF_138_ and SRPP_117_ have only a brief N- and C-terminal sequence flanking the REF domain, while REF_258_ has the longest N-terminal sequence, consisting of about 120 aa and containing a sequence similar to the β-subunit of ATPase (Figure 1C). Evolutionary analysis of the six protein members of REF and SRPP revealed that REF_138_, REF_175_, REF_258_, and SRPP_117_, SRPP_204_, SRPP_243_ are spread across the three major branches of the evolutionary tree, and REF_258_ is close to REF_175_ in the evolutionary relationship (Figure 1B).

### 2.2. Detection of the Six REF and SRPP Genes in H. brasiliensis Genomic DNA

**Figure 2:**
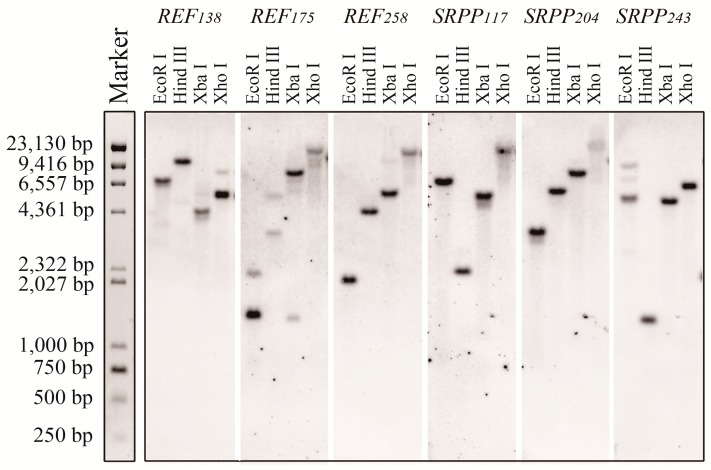
Southern blot of REFs and SRPPs in genomic DNA isolated from rubber tree clone RY 7-33-97. Endonucleases *Eco*R I, *Hind* III, *Xba* I, and *Xho* I were used for digestion of genomic DNA. DNA molecular mass markers are shown on the left.

The six REF and SRPP members are quite different from each other (Figure 1A). Southern hybridization was used to identify whether there are additional homologies of the six *REF* and *SRPP* genes in the genome of *H. brasiliensis*. Partial sequences of each REF or SRPP were used as probes, and endonuclease *Eco*R I, *Hind* III, *Xba* I, and *Xho* I were used for genomic DNA digestion. The results showed that only one band was visible hybridization for most of the endonuclease-digested products of *REF_138_*, *REF_258_*, *SRPP_117_*, *SRPP_204_*, and *SRPP_243_* (Figure 2), suggesting that *REF_138_*, *REF_258_*, *SRPP_117_*, *SRPP_204_*, and *SRPP_243_* have no other close homologs in the rubber genome. Two bands were visible upon *REF_175_* hybridization for most endonuclease-digested products (Figure 2), suggesting that there is one close homolog to *REF_175_*.

### 2.3. Subcellular Localization of REF and SRPP in Arabidopsis Protoplasts

**Figure 3:**
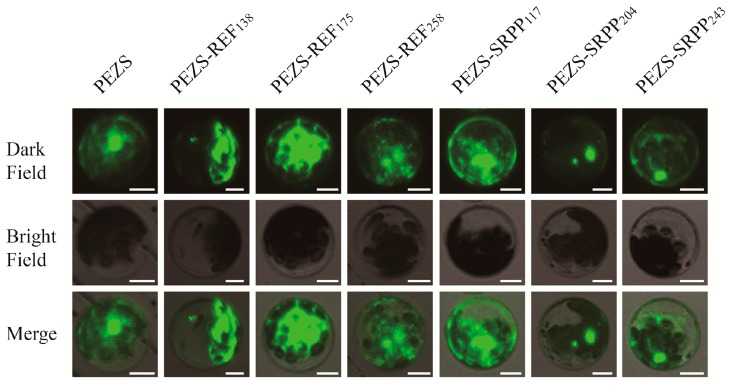
Subcellular localization of REF and SRPP proteins in *Arabidopsis* protoplasts using GFP-fluorescence. PEZS plasmids containing *REF-GFP* or *SRPP-GFP* fusions were transfected into the protoplasts. As a control a PEZS vector (containing GFP alone) was transfected. REF proteins appear to be gathered in the shadow zone of the cell under the view of Bright Field, and SRPP proteins appear to have diffused GFP fluorescence throughout the cells. GFP was excited with an argon laser at 488 nm and fluorescence images were taken using a confocal laser scanning microscope. Scale bars = 10 μm.

The 14.6 kDa REF_138_ protein is integral to the rubber particle membrane, and 24 kDa SRPP_204_ adheres to the membrane of rubber particles [16], and 19.6 kDa REF_175_ and 27.3 kDa REF_258_ are more tightly bound to the membrane than REF_138_ [20]. Transient expression of REF/SRPP-GFP in *Arabidopsis* protoplasts have been used to investigate if the subcellular localizations of these REF and SRPP proteins are similar to each other. The locations of REF and SRPP proteins in mesophyll cells of *Arabidopsis* leaves are not exclusive, as judged from the green fluorescence protein (GFP) fluorescence, but present two different tendencies for cellular location. The REF proteins demonstrated concentrate GFP fluorescence under shadow region observed in bright field by confocal which consisted of multiple organelles such as plastids. In contrast, the SRPP proteins showed diffused GFP fluorescence throughout the whole cells in cytoplasm, nuclear, and plasma membrane. Additionally, REF_258_ and the three SRPPs also demonstrated a kind of dotted pattern of GFP fluorescence in cytoplasm (Figure 3). The results revealed that in mesophyll cells REF_175_ and REF_258_ conducted a similar subcellular location as REF_138_, while SRPP_117_ and SRPP_243_ are located in a similar region as SRPP_204_, suggesting that they might have similar locations in laticifers. In addition, the dotted pattern for REF_258_, SRPP_117_ and SRPP_243_ is similar to the location of AtSRPPs/LDAPs in mesophyll cells in a certain way, which also confined to the cytosolic fraction with dot-like structures, and probably localized in lipid droplets [21,22].

### 2.4. Gene Expression Patterns of REF and SRPP in Rubber Latex under Ethylene Treatment

**Figure 4:**
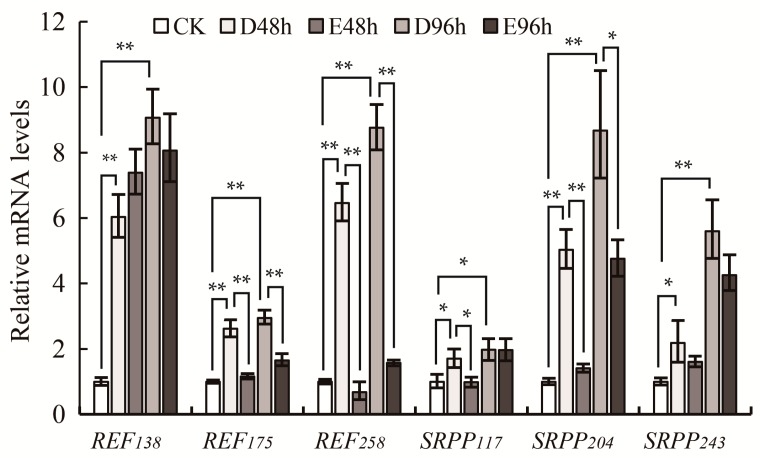
The expression patterns of *REF* and *SRPP* in the latex of rubber tree clone RY 7-33-97. The relative expression levels of the six *REF* and *SRPP* genes were normalized to *HbActin*. CK (control check), latex from the first tapping; D48h, D96h, latex harvested 48 and 96 h after tapping, distilled water treatment; E48h, E96h, latex harvested 48 and 96 h after tapping, ethylene treatment. Data from qRT-PCR contains three biological repeats and the error bars indicate the standard deviations. Above an error bar, the symbol * indicates a difference of *p* < 0.05 (Student’s *t*-test), and the symbol ** indicates a difference of *p* < 0.01 (Student’s *t*-test); D48h and D96h were compared to CK, and E48h and E96h were respectively compared to D48h and D96h.

Ethylene stimulation strongly promotes formation of fresh latex and increases latex dry matter yield [23]. Based on studies of *REF_138_* and *SRPP_204_*, some researchers suggested that ethylene stimulation did not change the expression levels of *REF* and *SRPP* in *H. brasiliensis* [19], but others suggested that ethylene treatment had a direct and positive effect on induction of *REF* gene expression [24]. We characterized the response to ethylene of the expression of the six *REF* and *SRPP* genes including *REF_138_* and *SRPP_204_*. Our data reveals that the six *REF*/*SRPP* genes showed a similar expression pattern, that they were increased by tapping and decreased or little influenced by ethylene (Figure 4). Among them, *REF_258_* and *SRPP_204_* displayed the significant changes upon both tapping and ethylene, while *REF_138_*, *SRPP_117_* and *SRPP_243_* had little changes upon ethylene stimulation (Figure 4).

### 2.5. Identification of Protein Species of Each REF/SRPP Protein

**Figure 5:**
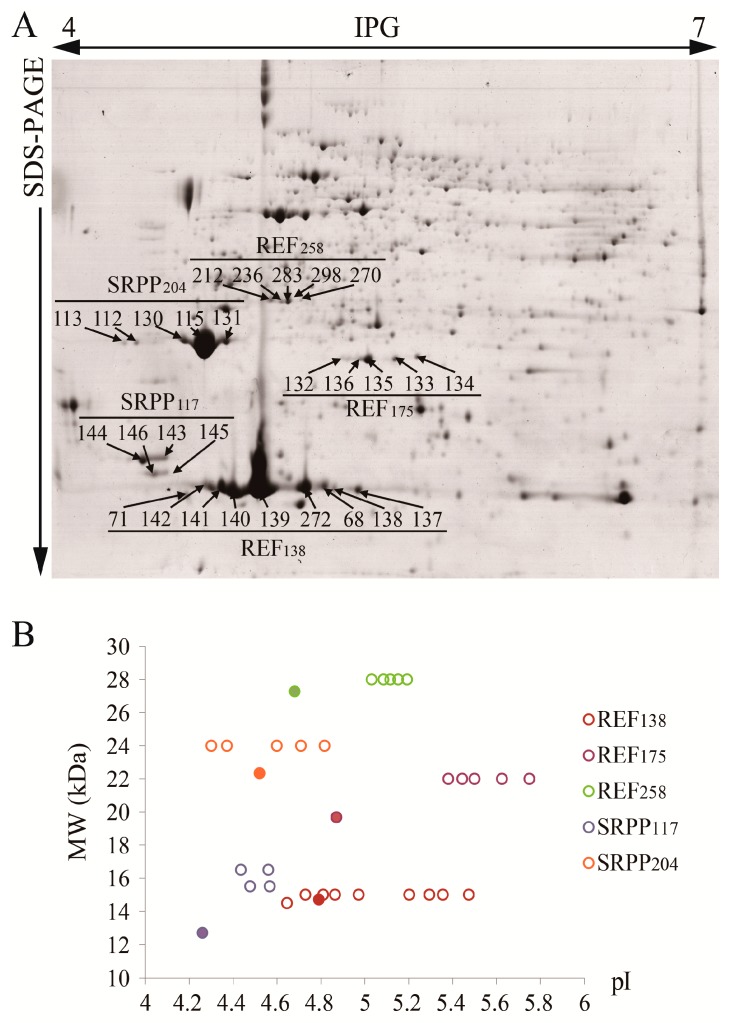
The REF and SRPP protein species. (**A**) The distributions of REF and SRPP protein species on a typical 2-DE gel of latex proteins from rubber tree RY 7-33-97 are presented; (**B**) The distribution of MW (molecular weight) and pI (isoelectric point) of each REF and SRPP protein species. The filled circles represent the theoretical MW and pI of each REF and SRPP protein, and the hollow circles represent the experimental MW and pI of each REF and SRPP protein species.

In a previous work on latex proteomics, we identified some protein spots as protein species of REF and SRPP, and noted that some of them were phosphorylated [23]. However, at that time the database used for identification of protein spots only included five REF/SRPP proteins, and the analysis did not distinguish these protein spots of individual REF and SRPP. To more comprehensively delineate the protein species of each REF and SRPP, we fractionated latex protein extracts using 2-DE and identified the protein species of each of the six abundant REF/SRPP proteins (Figure 5 and Table S1).

By searching the resultant mass spectrum (MS) data with a self-built database of the REF/SRPP proteins, a total of 28 protein spots were positively identified as five REF/SRPP proteins (Figure 5A), and SRPP_243_ was not matched to any protein spots on the 2-DE gel, probably due to its isoelectric point (pI) is beyond the separation range of the 4–7 immobilized pH gradient (IPG) strip used in the experiments. Multiple protein species identified as a particular REF or SRPP appeared to be much different in pI but not in molecular weight (MW). Among them, REF_138_ was the most abundant REF/SRPP protein, and had at least 9 protein species. Spots identified as REF_138_, REF_258_ or SRPP_204_ are distributed around their theoretical MW, while spots identified as REF_175_ and SRPP_117_ are clearly bigger than their theoretical MW (Figure 5B). The pI of protein species of an individual REF (or SRPP) was quite diverse. For example, the pI of the nine protein species of REF_138_ were distributed from pI 4.65 to 5.4, compared to the theoretical pI of 4.79 (Figure 5B).

### 2.6. Characterization of REF/SRPP Protein Species in Different Rubber Tree Clones in Response to Tapping or Ethylene Treatment

**Figure 6:**
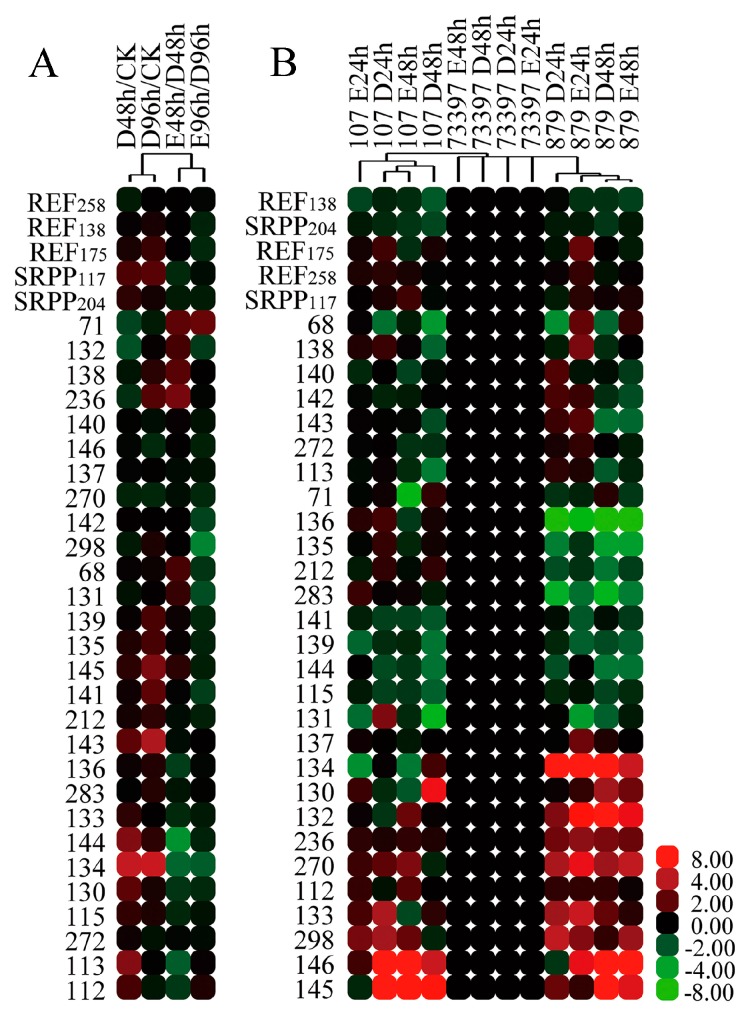

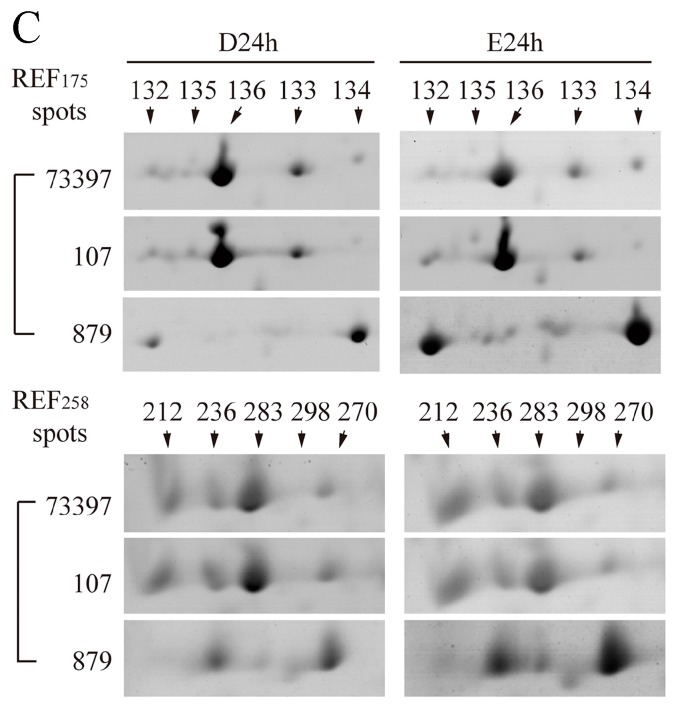
The abundance of the protein species of each REF/SRPP in high yielding and lower yielding clones of rubber tree upon ethylene treatment. (**A**) Clustering analysis of the relative abundance of the total protein and its protein species of each REF/SRPP after tapping and ethylene treatment. The abundance of each protein species in different treatments was normalized to their corresponding values in the control, CK; (**B**) Clustering analysis of the relative abundance of the total protein and the protein species of each REF/SRPP in rubber tree clones RY 7-33-97, PR 107 and RY 8-79. The abundance values of each protein or protein species were normalized to their corresponding values in RY 7-33-97; (**C**) 2-DE gel images of the protein species of REF_175_ and REF_258_ in rubber clones RY 7-33-97, PR 107 and RY 8-79. CK, the latex collected from the first tapping. D24h, D48h, D96h, latex from rubber trees at 24, 48, and 96 h under distilled water treatment. E24h, E48h, E96h, latex from rubber trees at 24, 48, and 96 h the under 3% ethephon treatment.

To analyze the influence of tapping and ethylene on the accumulation of these REF/SRPP protein species, total latex was collected from *Hevea* clone RY 7-33-97 before the treatments, and 48 and 96 h after treating with ddH_2_O (control) or with 3% ethephon. The total protein abundance of each REF/SRPP was calculated by adding all the abundance of their protein species together. The total abundance of REF/SRPP proteins was up-regulated or little changed after tapping, and down-regulated or little changed after ethylene treatment (Figure 6A), consistent with their gene expression patterns (Figure 4). The abundance of multiple protein species of a same REF/SRPP was diverse from each other. Most of these protein species were up-regulated by tapping, but down-regulated by ethylene. Spot 134 of REF_175_ is the most representative one, which is significantly up-regulated by tapping and down-regulated by ethylene. Only a few of these protein species were up-regulated by ethylene, such as spot 71 of REF_138_, spot 236 of REF_258_ (Figure 6A).

The abundance of different protein species of individual REF and SRPP was also determined in three different rubber tree clones, which have different latex productivity followed the order of RY 8-79 > RY 7-33-97 > PR 107 [25,26,27]. The total protein abundance of these REF/SRPPs showed small difference, but the abundance of individual protein species of them was very different among the three rubber tree clones (Figure 6B). Compared to RY 7-33-97 and PR 107, spots 132, 134, 236 and 270, had an obviously higher abundance, whereas spots 135, 136, 212 and 283 were extremely lower in RY 8-79 (Figure 6B). These eight protein species were all originating from REF_175_ and REF_258_ (Figure 6C), suggesting the two proteins are the most diverse REF/SRPP members among the three different rubber tree clones. The protein species 132, 134 of REF_175_ and 236, 270 of REF_258_ were not only predominant but also quite promoted by ethylene treatment in the high latex productivity rubber clone RY 8-79 (Figure 6C), suggesting the possible positive connection of these four protein species to latex productivity.

## 3. Discussion

### 3.1. The REF/SRPP Family in H. brasiliensis

Plant REF/SRPP family consists of either rubber biosynthesis related REF/SRPP proteins in latex-producing plants or some stress-related proteins in non-latex producing plants. All protein members in this family have a similar ~110 aa region called REF domain, but the differences between REF and SRPP subfamily have not been defined yet. In this work, the difference of REF and SRPP protein subfamily has been defined: REF subfamily members have a changeable N-terminal and a relatively short C-terminal beyond REF domain, while SRPP subfamily members have a short N-terminal and a changeable C-terminal (Figure 1A*). In H. brasiliensis*, through genome sequencing, eighteen predicted coding sequence (CDS) sequences for REF/SRPP were recently discovered, and these REF/SRPPs were annotated as eight REFs and ten SRPPs [17], most of these REF/SRPP sequences are unconfirmed by mRNA or proteins yet. In our previous work, we found only 13 of the 18 predicted REF/SRPP proteins could be identified in rubber latex by shotgun analysis [18]. In some other lactiferous plants, also are many REF/SRPP like proteins, which have demonstrated function in rubber biosynthesis. As in *Taraxacum brevicorniculatum*, six REF/SRPPs have been discovered, in which, SRPP 3, 4, and 5 are more close to REF of *Hevea*, and SRPP 2 is more close to SRPP of *Hevea* [28].

### 3.2. REF_258_ Is a Multiple-Domain Protein in REF/SRPP Family

The six latex abundant REF/SRPP protein genes are corresponding to *REF1* (*REF_138_*), *REF3* (*REF_175_*), *REF8* (*REF_258_*), *REF7* (*SRPP_117_*), *SRPP1* (*SRPP_204_*), and *SRPP2* (*SRPP_243_*) of the genome predicted CDS [28]. The *REF8* and *REF_258_* are not completely consistent, that predicted *REF8* misses a 108 bp sequence compared to *REF_258_*, which causes the protein of REF8 misses a 36 aa peptides in the N-terminal. We blasted REF_258_ in NCBI protein database, and identified a ~120 aa domain in the N-terminal as a β subunit of ATP synthase (Figure 1C). The N-terminal region has some similarity to Mycobacterial beta/delta fusion protein, and to a subunit from a *Methanosarcina barkeri* protein, which is with sodium-translocating ATP synthase.

We searched proteins with both REF domain and other extra domain like REF_258_ in the NCBI database, and noticed that this multiple domain architecture also existed in many other proteins such as Trehalase-REF (gi|596158908|XP_007222867, gi|645230894|XP_008222142, gi|658009949|XP_008340200), TM_PBP1_branched-REF (gi|460403916|XP_004247432, gi|565387203|XP_006359392), and MCP_signal-REF (gi|495337605|WP_008062341) (Figure S1). Unfortunately, there is no functional study of these proteins. In a multiple domain protein, each domain may fulfill a function independently or in concert with its neighbors [29]. REF is important for rubber biosynthesis, and the REF protein can aggregate with itself or with SRPP [15,30]. REF also has been shown to display strong aggregating properties with native neutral lipids extracted from *H. brasiliensis* latex [31]. In addition, REF_175_ and REF_258_ have been demonstrated bind more tightly to rubber particles than REF_138_ [20]. We speculated that REF_258_ could form complexes with other REF and SRPP proteins, and even other ATP synthase subunits, providing a new cooperative function of rubber biosynthesis and ATP usage. Such as REF-like proteins in avocado (*Persea americana*), which have revealed two aspects of functions: the packaging of triacylglycerols as well as oil biosynthesis on the oil body surface in mesocarp cells [32].

### 3.3. The Multiple Protein Species of REF/SRPP in H. brasiliensis

Ethylene stimulation increases the production of latex [33]. Studies showed ethylene stimulation not only accelerated the glycolytic pathway, which supplies precursors for the biosynthesis of IPP and natural rubber [34], but also activated the general biosynthesis of natural rubber between tappings [23]. The gene expression of most *REF/SRPPs* decreased (Figure 4), and the total protein abundance of each REF/SRPP was slightly changed after ethylene treatment (Figure 6A). At the same time, the abundance of multiple protein species of each REF/SRPP changed diversely among the three rubber tree clones with different latex productivity (Figure 6B). Multiple protein species of a protein could be created through single-nucleotide polymorphisms (SNPs), alternative splicing (AS), post translational modifications (PTMs) or protein degradation, but more than 90% of them are generated by PTMs [35]. In our previously work, spots 140/141/142, 144/146, 115/130 were found to be phosphorylated modifications on REF_138_, SRPP_117_, and SRPP_204_, respectively [23]. In this experiment, none of these phosphorylated modifications changed much after ethylene treatment (Figure 6A), or showed obvious difference among different rubber clones (Figure 6B). However, we found some protein species of REF_175_ (132, 134) and REF_258_ (236, 270) were not only predominant but also ethylene-responsive in high latex productivity clone RY 8-79 (Figure 6C), suggesting their positive relations to latex production. These four protein species of REF_175_ and REF_258_ seem not to be dyed in red by Pro-Q diamond [23], suggesting they are not phosphorylated protein species, still their modifications were unknown. Protein species of a same REF/SRPP varied diversely in response to ethylene treatment or among rubber tree clones might have biological significance. We proposed specific protein species which are dominant in high latex productivity rubber tree and positive response to ethylene stimulation might be important for natural rubber biosynthesis.

## 4. Materials and Methods

### 4.1. Plant Materials and Treatments

Latex samples were obtained from newly tapped mature rubber plants (~8-year-old, *H. brasiliensis* Mull. Arg., clones RY 7-33-97, RY 8-79 and PR 107) growing at an experimental farm of the Chinese Academy of Tropical Agricultural Sciences at Danzhou city in Hainan province of China. Ethephon is a type of ethylene release agent which is capable of direct chemical decomposition into ethylene under physiological conditions [36,37]. When ethephon is applied to the bark in the region of the tapping cut, ethylene commences immediately at the site of application, and quickly translocates throughout the plant [38]. To determine the direct effects of ethylene stimulation, rubber trees were tapped one day before treatments, then the tree cuts were brushed with ethephon (3%, *v*/*v*) or ddH_2_O (the control) 24 h after the first tapping as described [39]. Latex samples were collected from each rubber tree one day before treatment (as the control, CK) and the second (D24h, E24h), third (D48h, E48h) and fifth (D96h, E96h) day after ethephon or ddH_2_O treatments. Treatments and sample collections were carried on with three independent biological replicates and latex from 15 trees were harvested for the 3 replicates (5 trees/replicate). Among these clones, RY 7-33-97 and RY 8-79 are the two elite clones bred by Chinese Academy of Tropical Agricultural Sciences, the former one is a fast-growing and high-yielding clone, and the later one is an early maturing and high-yielding clone. PR 107 is a late-maturing clone, having lower yield in its first few years, and gradually increases in later years. These three clones have remarkable differences in their latex production ability in the first few years of tapping, the latex yield being RY 8-79 > RY 7-33-97 > PR 107 [25,26,27].

### 4.2. Gene Clone

RNA extraction from the latex was performed using high SDS extraction buffer (100 mM Tris-HCl, 300 mM LiCl, 10 mM EDTA-Na_2_, and 10% SDS). The first strand cDNA was synthesis using 1 μg total RNA and with SMARTer™ RACE cDNA Amplification Kit (Clontech, Dalian, China). The PCR products of six REF/SRPP genes were amplified and separated through an agarose gel and purified with the AxyPrep™ DNA gel extraction kit (Axygen, Suzhou, China). Purified PCR products were ligated into a clone vector PMD18T (Takara, Dalian, China) and transformed into *Escherichia coli* strain JM109. Multiple clones from each were sent for sequencing (Life Biological Technology Co., Guangzhou, China).

The complete cDNA sequence of REF and SRPP were submitted to the GenBank database under accession numbers of REF_138_ (KR076812), REF_175_ (KR076813), REF_258_ (KR076814), SRPP_117_ (KR076815), SRPP_204_ (KR076816), and SRPP_243_ (KR076817). The MW and pI of the deducted proteins from these genes were calculated using DNAMAN 5.0 software (Lynnon Biosoft, San Ramon, CA, USA). Multiple sequence alignments of REF and SRPP were assembled using the EBI ClustalW [40] and visualized by BioEdit 7.0 software (Ibis Biosciences, Carlsbad, CA, USA). Phylogenetic and molecular evolutionary analyses were conducted using MEGA version 5 software as described [41].

### 4.3. Southern Blot Analysis

Southern blotting was conducted by using Dig Application Manual for Filter Hybridization (Roche, Basel, Switzerland). *H. brasiliensis* genomic DNA was extracted from the leaves of rubber tree and digested with *Bam*H I, *Eco*R I, *Hind* III, and *Xba* I (Thermo Scientific, Guangzhou, China). Ten μg of each digestion product was electrophoresed on a 0.8% agarose gel at 1 V/cm overnight, and then transferred onto a positively charged nylon membrane (Roche, Basel, Switzerland) through capillary transfer by 20× SSC (saline sodium citrate) for about 12 h. UV (ultraviolet) crosslink of the DNA to the filter was at a strength of 90,000 μJ/m^2^. The cross-linked filters were hybridized with ~350 bp Dig PCR labeled probes of each *REF* and *SRPP* genes, respectively. The primers for the probes are listed in Table S2. Hybridization was performed at 42 °C and the probes were washed at a low stringent condition. DNA blots were visualized by LAS4000mini (GE Healthcare, Uppsala, Sweden).

### 4.4. REF/SRPP-GFP Fusion Transient Expression

The sequences of the *REF/SRPP* genes were amplified by PCR and cloned into the vector, pEZS-NL, which has a GFP tag positioned at the C-terminal of the insert. The purified plasmids were then sequenced to confirm the fusion construct. The plasmids were extracted by an endotoxin free plasmid extraction kit (CWBIO, Shanghai, China) to a final concentration of 2 μg/μL, and stored in −20 °C.

Transient expression assay was performed according to the method [42]. Mesophyll protoplasts were isolated from the leaves of 3-week old *Arabidopsis* Col-0 plants. Ten μg of each *REF*/*SRPP-GFP* fusion plasmids were transfected into 4 × 10^4^ protoplasts using polyethylene glycol (PEG) solution (0.4 g/mL PEG 4000, 0.2 M mannitol, 100 mM CaCl_2_), incubated at room temperature for 20 min, washed and re-suspended with 1 mL W5 solution (154 mM NaCl, 125 mM CaCl_2_, 5 mM KCl, 2 mM MES, 5 mM glucose at pH 5.7), placed under a dark condition at 22 °C for 16–24 h, then imaged by confocal laser scanning microscope (Olympus fv1000, Tokyo, Japan).

### 4.5. Quantitative PCR Analysis of REF and SRPP

The first strand cDNA synthesis was performed using 1 μg of total RNA and a Revert Aid First Strand cDNA Synthesis Kit in 25 μL volume (Thermo Scientific, Guangzhou, China). One μL of each cDNA synthesis was used to amplify the sequence for each REF or SRPP. The primers used for PCR analysis were designed according to the sequence dissimilarity regions among the six *REF/SRPP* genes. The *HbActin* gene (JF775488.1) from *H. brasiliensis* was used as an internal control. All primers are listed in Table S2. For quantitative PCR (qPCR), the amplifications were performed using 2× Maxima SYBR Green/ROX, qPCR Master Mix (Thermo Scientific, Guangzhou, China) and the Mx3005P QPCR System (Agilent Technologies, Santa Clara, CA, USA). The relative mRNA levels were calculated by MxPro, the system included software based on 2^−ΔΔ*C*t^ method [43]. Three biological replicates were performed in qPCR with samples from the same batch of latex of RY 7-33-97 as was used for protein extraction.

### 4.6. Protein Extraction and 2-DE

Latex proteins were extracted as described [39]. Protein concentration was determined by the Bradford assay with BSA as a standard. For 2-DE, 1300 μg proteins were loaded onto the 24 cm, pH 4–7 linear IPG strips (GE Healthcare, Uppsala, Sweden), then performed isoelectric focusing and SDS-PAGE as described [31]. The MW, pI, and volume% of each protein spot was calculated by ImageMaster 5.0 software (GE Healthcare, Uppsala, Sweden). Spots were eluted from gel for protein enzymolysis and identification through mass spectrometry on an AB 5800 MALDI-TOF/TOF-MS instrument (AB SCIEX, Foster City, CA, USA) as described [44]. The sequences of REF/SRPP were formatted into text files that can be used by ProteinPilot software to provide identification of the 2-DE spots. The values of the vol.% were used to represent the level of each REF/SRPP protein. The total abundance for each REF/SRPP protein was calculated by adding the abundance of its spots (protein species) together. The clustering of REF/SRPP protein species that result from different treatments or different rubber tree clones were performed by Cluster 3.0 software and visualized by TreeView1.1.6 software (Stanford University, Stanford, CA, USA).

## 5. Conclusions

In this study, we systematically characterized the sequences, locations, expressions, and protein species of the latex-abundant REF/SRPP family members in *H. brasiliensis*. Based on the obtained results, we further defined the characteristics of REF and SRPP subfamily, clarified the expression patterns of REF/SRPP members upon ethylene treatment, and disclosed the multiple protein species of each REF/SRPP protein. The finding of REF domain and ATPase β subunit united in REF_258_ implies a coordinating role of REF_258_ in latex production. More importantly, we noticed that the protein species of each REF/SRPP protein behaved differently among the three rubber tree clones RY 7-33-97, RY 8-79 and PR 107, and REF_175_ and REF_258_ each has two protein species predominant and ethylene-responsive in high productivity rubber tree clone RY 8-79. The results suggested that only individual protein species of REF/SRPP protein could positively relate to rubber biosynthesis and latex production. We presented some tips for further study of the not-yet explicit process of rubber biosynthesis.

## Supplementary Materials

Supplementary materials can be found at www.mdpi.com/1422-0067/18/5/958/s1.
